# Emerging Therapeutic Options for Refractory Pulmonary Sarcoidosis: The Evidence and Proposed Mechanisms of Action

**DOI:** 10.3390/jcm13010015

**Published:** 2023-12-19

**Authors:** Nathaniel C. Nelson, Rebecca Kogan, Rany Condos, Kerry M. Hena

**Affiliations:** Division of Pulmonary, Critical Care, and Sleep Medicine, Department of Medicine, New York University, 301 E 17th St Suite 550, New York, NY 10003, USA

**Keywords:** sarcoidosis, refractory, therapy, granuloma, fibrosis

## Abstract

Sarcoidosis is a systemic disease with heterogenous clinical phenotypes characterized by non-necrotizing granuloma formation in affected organs. Most disease either remits spontaneously or responds to corticosteroids and second-line disease-modifying therapies. These medications are associated with numerous toxicities that can significantly impact patient quality-of-life and often limit their long-term use. Additionally, a minority of patients experience chronic, progressive disease that proves refractory to standard treatments. To date, there are limited data to guide the selection of alternative third-line medications for these patients. This review will outline the pathobiological rationale behind current and emerging therapeutic agents for refractory or drug-intolerant sarcoidosis and summarize the existing clinical evidence in support of their use.

## 1. Introduction

### 1.1. What Is Sarcoidosis?

Sarcoidosis is a multisystem disease of unknown etiology characterized by non-caseating granulomatous inflammation in affected organs, most commonly in the lung and intrathoracic lymph nodes [1]. The natural history of pulmonary sarcoidosis varies, ranging from asymptomatic interstitial involvement to advanced fibrotic lung disease. Not surprisingly, treatment indications and responses mirror the disease’s clinical heterogeneity [2]. 

### 1.2. Pathobiology of Granuloma Formation

The compact, non-necrotizing epithelioid granuloma, which is the pathologic hallmark of sarcoidosis, has been well described [3,4,5,6]. In the lung, the granulomas of sarcoidosis are classically found in a lymphatic distribution along bronchovascular bundles. They are primarily comprised of activated macrophages, multinucleated giant cells, mononuclear cells, and lymphocytes. In the acute stages of granuloma development, they exhibit a highly polarized expression of T helper Type 1 (Th1) cytokines, including interferon-gamma (IFN-γ) and tumor necrosis factor-alpha (TNF-α), along with evidence of Th17 cell signaling [7,8]. While most disease either remits spontaneously or proves responsive to treatment with corticosteroids, approximately one third of patients experience persistent or recurrent inflammation [9,10]. The mechanisms underlying the progressive fibrotic phenotype remain an area of research and have been hypothesized to reflect a transition from Th1- to Th2-mediated pathways [11,12,13]. 

### 1.3. Who Needs Treatment? 

Most patients with pulmonary sarcoidosis either do not require treatment or respond to first-line corticosteroids. Deciding whom to treat, how to adjust therapy, and which therapies to choose for patients with progressive or refractory disease remain crucial questions of significant clinical relevance [14,15,16,17]. Ideally, the decision to initiate, maintain, discontinue, resume, or escalate therapy should be based on evidence of active granulomatous inflammation, with a resulting physiological or functional impairment impacting the patient’s quality-of-life [18]. Due to limited randomized controlled clinical trials, expert consensus provides much of the guidance in sarcoidosis management [19]. Key treatment concepts emerged from a 2020 international conference of experts participating in a Delphi process, which resulted in published guidelines advocating for therapy escalation or adjustment based on disease severity, progression from acute to chronic phenotypes, or pharmacologic toxicity. While the expert panel achieved consensus regarding indications for adding a third-line biological agent, specifically TNF-inhibitors, no additional consensus was reached for therapeutic options for refractory disease. To date, corticosteroids and repository corticotropin injection remain the only medications with Food and Drug Administration (FDA) approval for the treatment of sarcoidosis [19].

### 1.4. What Is Considered Refractory Sarcoidosis 

**Figure 1:**
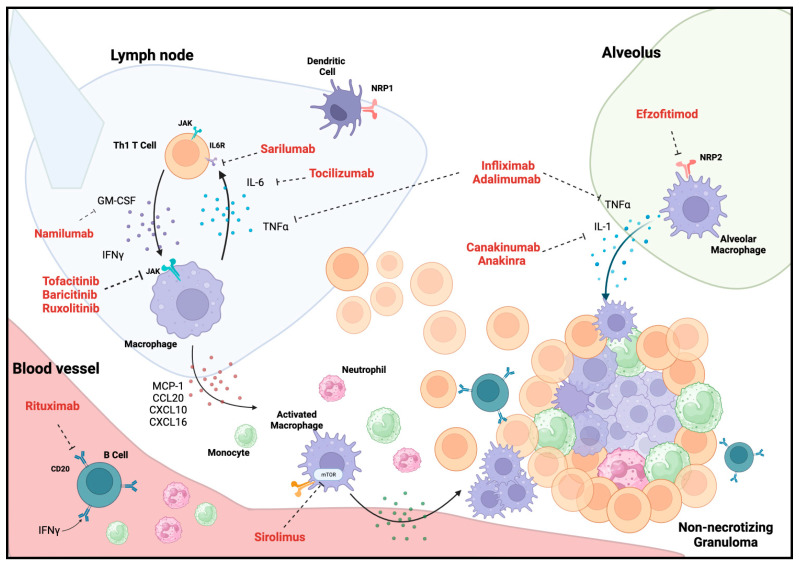
Schematic for non-necrotizing granuloma formation in pulmonary sarcoidosis highlighting emerging therapeutic targets and medications. Abbreviations: CCL20—chemokine ligand 20; CXCL10—C-X-C ligand 10; CACL16—C-X-C ligand 16, GM-CSF—granulocyte-macrophage colony-stimulating factor, IFN-γ—interferon-gamma, IL—interleukin; IL6R—interleukin-6 receptor, JAK—janus kinase; mTOR—mammalian target of rapamycin; MCP-1—monocyte chemoattractant protein-1; NRP—neuropilin; TNFα—tumor necrosis factor-alpha. Created with BioRender.com.

While there is no consensus or data-driven definition of refractory sarcoidosis, several have been proposed. Most suggested definitions include a situation in which second-line treatments (methotrexate, azathioprine, leflunomide, antimalarials, or mycophenolate mofetil) prove to be insufficient to achieve clinical remission with corticosteroid dosing under 10 mg per day (prednisone equivalents) [20]. An obvious challenge in the treatment of refractory pulmonary sarcoidosis is a lack of clear understanding of the pathobiological processes that have resulted in the disease and its resistance to therapy. This review will address the emerging potential therapeutic modalities for refractory pulmonary sarcoidosis, with a focus on their proposed biologic role (Figure 1) in mitigating granulomatous inflammation or pro-fibrotic pathways, available evidence, and salient clinical parameters to monitor for efficacy or harm (Table 1). 

## 2. Treatment Options for Refractory Sarcoidosis

### 2.1. TNF-Inhibitors

#### 2.1.1. Rationale

TNF-α is a pro-inflammatory cytokine with a broad range of biological activities. Initially recognized in the 1970s for its ability to induce hemorrhagic necrosis of transplantable tumors in murine models, it was later identified as a catabolic hormone mediating anorexia and weight loss in chronic disease (and, thus, briefly named “cachectin”) [21]. It has since been shown to play a critical role in directly and indirectly promoting the release of many inflammatory mediators in both acute and chronic disease. Interest in its potential as a therapeutic target for sarcoidosis was solidified after trials demonstrated its efficacy in other chronic inflammatory conditions, including rheumatoid arthritis and Crohn’s Disease [22].

A series of experiments beginning in the 1990s demonstrated an association between TNF-α and active sarcoidosis. Müller-Quernheim and colleagues measured TNF-α and interleukin-1 (IL-1) spontaneously produced by alveolar macrophages (AM) cultured from the bronchoalveolar lavage (BAL) fluids of 43 patients with sarcoidosis [23]. They observed a significant increase in both cytokines released by AMs from patients with clinically active disease (defined by progressive symptoms, pulmonary function impairment, or radiographic involvement) compared to inactive disease or healthy controls. Interestingly, a corresponding increase in TNF-α and IL-1 measured in peripheral blood monocyte cells (PBMC) was not observed, supporting a model of “compartmentalized” inflammation at the site of the affected organ. Zheng and colleagues reproduced this finding and demonstrated a correlation between TNF-α and percentage CD4+ (cluster of differentiation) lymphocytes, suggesting that this may serve as a surrogate marker for disease activity [24]. Müller-Quernheim’s group subsequently demonstrated that patients without a clinical indication for treatment at initial assessment were at higher risk of disease progression if they were found to have elevated AM production of TNF-α at baseline [25]. 

TNF-α is known to play an integral role in maintenance of granulomas during infection with *Mycobacterium tuberculosis,* which it does via chemokine induction, immune cell recruitment, and facilitating leukocyte aggregation [26,27]. Among patients with sarcoidosis, TNF-α has also been observed to upregulate intracellular adhesion molecule-1 (ICAM-1) expression on AMs, which is believed promote granuloma formation [28]. A meta-analysis found that *TNF* promotor polymorphism is associated with sarcoidosis [29]. It is, therefore, widely hypothesized that TNF-α plays a key role in granuloma formation and persistence in sarcoidosis, and by blocking this cytokine, the disease may be attenuated. 

#### 2.1.2. Clinical Evidence

The first trial to evaluate a TNF inhibitor (etanercept) in pulmonary sarcoidosis was terminated early due to treatment failure [30]. This was in contrast to its amelioration of cutaneous and joint manifestations in earlier case series and case reports [31]. Discrepancies in efficacy might be explained by differences in the targeting of the TNF-α molecule. Unlike other TNF-inhibitors, which are monoclonal antibodies that bind to both soluble and membra-bound TNF, etanercept is a soluble receptor construct that can only bind to soluble TNF [32]. Nevertheless, interest in the therapeutic class persisted, and a retrospective case series of patients treated with infliximab subsequently demonstrated improvement in manifestations of corticosteroid refractory sarcoidosis, many with severe cutaneous or other extrapulmonary manifestations of disease [33]. The efficacy of infliximab for pulmonary sarcoidosis was later supported by a pair of randomized placebo-control trials published in 2006. The first of these trials detected a trend towards increased vital capacity in 18 patients with active stage II–IV pulmonary sarcoidosis, all of whom were either on at least three months of steroids, required alternative therapy due to suboptimal steroid response, or exhibited steroid intolerance [34]. The second trial enrolled 134 patients with chronic pulmonary sarcoidosis (also defined as having been treated for more than three months). In this trial, Baughman and colleagues found that infliximab (at 5 mg/kg) resulted in an increase in forced vital capacity (FVC) compared to placebo (mean percentage of predicted increase of 2.5% over 24 weeks) [35]. Improvement in FVC was greater among those with longer disease duration, lower baseline FVC, and higher Saint George Respiratory Questionnaire (SGRQ) scores. The study did not demonstrate any significant difference in secondary respiratory endpoints, including SGRQ, 6-minute walk distance (6MWD), or Borg dyspnea score. A retrospective analysis of data from this trial suggested that this effect may be most significant among patients receiving lower doses of maintenance corticosteroids and demonstrated diminished improvements in FVC among patients receiving maintenance doses ≥15 mg/d prednisone [36]. Additional sub-analysis of this cohort evaluated the effect of infliximab on extrapulmonary manifestations and showed an improvement during the 24-week trial period, with relapse during the subsequent washout period [37]. 

A recently published real-world analysis of infliximab used in 14 patients with previously refractory pulmonary sarcoidosis observed a treatment success of 78.6% (95% confidence interval, CI, 49.2–95.3). Treatment success was defined as either improvement or stability in measurements of FVC or forced expiratory volume in a one-second (FEV1) percentage of the predicted results [38]. The therapy proved even more efficacious for 6 patients with central nervous system (CNS) involvement and 12 patients with cutaneous disease, achieving 100% (95% CI 54.1–100%) and 91.7% (95% CI 61.5–99.8%) treatment success rates, respectively. 

In 2011, Kamphuis and colleagues published a case series consisting of five patients with chronically active pulmonary and extrapulmonary sarcoidosis treated with adalimumab [39]. Four of the five patients achieved radiographic improvement in thoracic lymphadenopathy, as well as subjective improvement in fatigue and dyspnea during a 12-week observational period. With increased dosing frequency, radiographical and symptomatic improvement were also achieved in the fifth patient. Sweiss and colleagues described an additional 11 patients with refractory pulmonary sarcoidosis treated with adalimumab over a 52-week study period [40]. During this time, four patients achieved a >5% increase in absolute FVC, five patients improved in terms of 6MWD, and nine noted improvement in Borg dyspnea scores. In 2016, Crommelin et al. evaluated the efficacy of adalimumab in 18 patients who had discontinued infliximab due to adverse events or lost efficacy in the setting of antibody formation [41]. Of these patients, 13 experienced stabilization or improvement in FVC and all exhibited stabilization or improvement in measurements of soluble IL2 receptor (sIL2R) over a 12-month treatment period. Despite these promising observational reports, a placebo-controlled trial of 16 subjects with pulmonary and cutaneous sarcoidosis demonstrated improvement in the skin lesion area with adalimumab but no significant difference in radiographical manifestations of pulmonary sarcoidosis or pulmonary function at the end of the study period [42]. However, the 12-week study period may have been too short in duration to evaluate for a potential positive or even negative effect of adalimumab on chest radiography or pulmonary physiology. 

To date, infliximab remains the only biological therapy promoted as a third-line treatment option for pulmonary sarcoidosis in the Delphi consensus recommendations [19].

#### 2.1.3. Adverse Effects and Clinical Monitoring 

Safety data from the early randomized control trials of infliximab in pulmonary sarcoidosis showed similar rates of serious adverse events in patients treated with infliximab compared to placebo during the study period (23.1% vs. 18.2%), with adverse events leading to discontinuation in 4.5% of the placebo group and 5.5% in the treatment group [35]. However, in a real-world assessment of infliximab used in patients with sarcoidosis, adverse events led to permanent discontinuation of the therapy in 20% of patients. The most commonly observed adverse event was pneumonia (18%), followed by leukopenia (15%) and infusion reaction (12%). Anaphylaxis was also observed in four patients (12%) [38]. 

Some case reports describe paradoxical adverse events (PAE) occurring during TNF-α inhibitor therapy, whereby patients developed the new onset of a disease that is typically improved by this class of medication. The most commonly described PAE is psoriasis [43,44]. The mechanism for these PAEs is uncertain, but hypotheses include an imbalance in cytokine production, the differential immunological properties between the monoclonal antibodies and TNF-α soluble receptor, an unopposed type I interferon production, and/or a shift towards a Th1/Th2 profile [45]. One large retrospective study of patients with spondylarthritis found no significant difference in PAEs among patients taking TNF-α inhibitors and those treated with conventional disease-modifying anti-rheumatic agents [46].

The reactivation of latent tuberculosis is a well-documented risk of treatment with TNF-inhibitors [47,48,49]. The reactivation of hepatitis is also a concern [50,51]. For this reason, pre-treatment testing for latent infections is generally recommended, particularly in patients with increased baseline risk. Clinical monitoring for all patients includes routine lab testing for leukopenia and transaminitis. 

### 2.2. Anti-CD20 

#### 2.2.1. Rationale

Sarcoidosis is generally considered to be a T-cell mediated disease. Nevertheless, it is associated with hypergammaglobulinemia, circulating immune complexes, and peri-granuloma infiltration of B-cells, which suggests a supporting role for humoral immunity in its pathogenesis [52,53]. One proposed mechanism for B-cell involvement is via the Th1 cell production of IFN-γ, which stimulates the B-cell activating factor (BAFF), an anti-apoptotic signal involved in B-cell differentiation [54]. Several studies have demonstrated increased BAFF activity in patients with active sarcoidosis compared to those with quiescent disease and healthy controls [55,56]. Patients with active sarcoidosis have also been shown to have distinct patterns of B-cell populations, with higher proportions of transitional B-cells and lower proportions of memory B-cells, though the clinical significance of this pattern is unclear [53,55].

The putative involvement of B-cells in the pathogenesis of sarcoidosis suggests a potential role for B-cell blocking agents as a therapeutic class in its treatment. Rituximab is a chimeric monoclonal antibody which, when bound to the transmembrane protein CD20, induces B-cell destruction via antibody and complement-mediated cytotoxicity, as well as the induction of apoptotic pathways [57].

#### 2.2.2. Clinical Evidence 

There are several case reports of patients with pulmonary sarcoidosis who have experienced clinical improvement with rituximab. In 2008, Belkhou and colleagues published their experience of treating a woman with sarcoidosis with moderate restrictive pulmonary disease and polyarthritis who remained steroid-dependent despite the addition of methotrexate. Three months after the initiation of rituximab, her PFTs had normalized and prednisone was discontinued [58]. In 2015, Cinetto and colleagues published a case series of three patients with pulmonary sarcoidosis with variable responses to rituximab [59]. The first patient was a man with pulmonary and extra-thoracic lymph node involvement who had previously proved responsive to corticosteroids and methotrexate; however, he had experienced progression when taken off therapy. He was intolerant of azathioprine and progressed on cyclophosphamide but experienced radiographical and symptomatic improvement with rituximab administered biweekly over 12 weeks, with sustained remission over an 18-month follow-up period. The second patient had pulmonary, extra-thoracic lymph node, and cutaneous disease, and he remained steroid-dependent despite treatment with azathioprine and methotrexate. He experienced symptomatic improvement and was able to discontinue steroids after three monthly infusions of rituximab (1 g) with concurrent hydroxychloroquine. He experienced relapse four months later, prompting a second course of rituximab with a less robust response. The third patient was a woman with pulmonary, cutaneous, and ocular disease who had exhibited improvement with various lines of therapy including TNF-inhibitors but experienced frequent exacerbations (primarily of her cutaneous and ocular manifestations) during periods of maintenance therapy. While the addition of rituximab to her regimen did not achieve remission in symptoms, subsequent re-treatment with infliximab achieved complete resolution of skin lesions and improvement in ocular disease. 

In a 2015 letter to the European Respiratory Journal, Sweiss and colleagues recounted the results of a prospective phase I/II clinical trial of rituximab in ten patients with at least two years of moderate-to-severe pulmonary refractory sarcoidosis with at least three months of ≥10 mg prednisone daily or any dose of prednisone with one or more steroid-sparing agent [60]. Participants received 1 g of rituximab at baseline and then again two weeks later. Treatment response was defined as a >5% absolute improvement in FVC and/or a >30 m increase in 6MWD. At 24 weeks, 5 out of 10 patients met the predicted endpoint of >5% absolute improvement in FVC percentage and 5 out of 10 patients had >30 m improvement in their 6MWD. However, disappointingly, at 52 weeks, only 2 out of 10 met the endpoint of FVC improvement and three of 10 exhibited improvement in 6MWD. Although this trial suggests that there may be a role for rituximab in treatment for refractory pulmonary sarcoidosis, more data are needed in order to determine whether it represents an efficacious treatment option. Moreover, the absence of a consistent administration schedule or dosage of rituximab, as well as co-treatment with different immune-suppressants in all of these studies, makes it difficult to compare results or translate them into clinical practice.

#### 2.2.3. Adverse Effects and Clinical Monitoring 

In a phase I clinical trial of 15 patients with B-cell lymphoma treated with 10–500 mg/m^2^ of rituximab, mild and moderate infusion-related side effects were observed, including nausea, headache, fever, chills, bronchospasm, and orthostatic hypotension. The most commonly observed side effect was fever during infusion, which was observed in 13 patients [61]. Observational data in cancer patients treated with rituximab corroborate the finding that adverse events are predominately mild or moderate in severity, typically occur during the first infusion, and do not usually recur with subsequent infusions [62]. The side effect profile has been observed to be lower among patients with lower levels of circulating B-cells prior to treatment, which may suggest that patients being treated for autoimmune conditions rather than B-cell lymphomas are at lower risk of reaction [57,61]. 

Several studies have demonstrated a rapid decrease in B-cells after rituximab infusion that typically persists for several months, placing patients at risk of infection. A 1998 prospective multicenter trial of rituximab in 166 patients with lymphoma reported 68 (41%) infections by the one-year follow-up period, with the majority being bacterial in origin. Most of these (61/68) were characterized as mild [63]. 

In 2019, Lower and colleagues performed a retrospective review of 2109 patients at a single center treated with the TNF-inhibitors or rituximab [64]. Rituximab had the lowest rate of discontinuation (29%) compared to infliximab (55%) and adalimumab (58%). The most common reason for the discontinuation of rituximab was the lack of treatment response, followed by insurance coverage and allergic reactions. No patients discontinued the drug due to infections during the study period. 

The reactivation of indolent infections is also a concern. Several retrospective case studies have not found any association between rituximab therapy and cases of tuberculosis in endemic regions [65,66]. The reactivation of hepatitis B with rituximab therapy has been documented, and this risk is likely higher in patients receiving rituximab with chemotherapy. There may be a role for the concurrent initiation of nucleoside analog in patients with a history of hepatitis B [67,68]. Appropriate immunizations must be provided several weeks prior to initiation [69]. In addition, accumulating evidence has implicated anti-CD20 therapy with a risk of severe outcomes related to COVID-19 infections, which must be considered in the current era [70]. 

Prior to initiating therapy, serum immunoglobulin levels and hepatitis serologies should be obtained [71]. Routine clinical monitoring generally includes evaluation for cytopenia, hypogammaglobulinemia, and infection. 

### 2.3. JAK-Inhibitors

#### 2.3.1. Rationale 

The Janus kinase/signal transduction and activator of transcription (JAK/STAT) signaling pathway plays an integral role in cell function and homeostasis and regulates the expression of key mediators of hematopoiesis, apoptosis, tissue repair, and inflammation [72]. Discovered in the 1990s through work that sought to outline how interferon triggers the expression of genes, the dysregulation of this pathway has been subsequently recognized in various pathologies, ranging from malignancies to autoimmune disease [73]. A number of cytokines believed to be key to macrophage activation and granuloma formation, including IFN-γ, IL-2, IL-4, and IL-23, are known to signal via this pathway, which is initiated by the binding of the cytokine or growth factor with its specific cellular transmembrane receptors, thus activating JAKs and enabling interaction with intracellular STAT proteins, which then travel to the nucleus to affect gene transcription. 

In 2009, a small study by Rosenbaum and colleagues demonstrated that messenger RNA (mRNA) transcripts associated with STAT signaling were upregulated in the peripheral blood, lung parenchyma, and thoracic lymph nodes of patients with sarcoidosis compared to healthy controls, with a marked increase in phosphorylated STAT1 transcripts observed within the granuloma [74]. A subsequent analysis of six patients with pulmonary sarcoidosis and six healthy control subjects demonstrated that genes differentially expressed in the lung tissue of patients with sarcoidosis were most closely related to the JAK/STAT signaling pathway [75]. In a slightly larger study, differentially expressed genes related to JAK/STAT signaling measured in the measured in PBMC of patients were shown to distinguish between healthy controls and pulmonary sarcoidosis, and, furthermore, they differentiated stable disease from that with a progressive or multiorgan involvement phenotype [76].

#### 2.3.2. Clinical Evidence 

In 2018, Damsky and colleagues published their experience of treating a patient with pulmonary and refractory cutaneous sarcoidosis with the small molecule JAK inhibitor tofacitinib [77]. The patient had Scadding Stage II disease with non-caseating granulomas evident on transbronchial lung biopsy but no significant respiratory symptoms. She had extensive indurated papules and plaques covering large portions of her scalp, neck, torso, arms, and legs, which had proven refractory to topical glucocorticoids, minocycline, hydroxychloroquine, methotrexate, adalimumab, tacrolimus, and apremilast (systemic glucocorticoids had been withheld due to comorbid diabetes and hypertension). The initiation of tofacitinib resulted in clinical and histological remission of her skin lesions, which recurred following the cessation of the therapy. 

Subsequent reports have described additional JAK inhibitors (ruxolitinib and barcitinib) and improvement in pulmonary sarcoidosis [78,79,80]. The results of an ongoing open-labeled trial (NCT03910543) evaluating the efficacy of tofacitinib in refractory cutaneous sarcoidosis included eight patients with active pulmonary sarcoidosis and one patient with active myocardial disease [81]. Internal organ disease activity was assessed via whole body positron emission tomography and computed tomography (PET-CT). Of eight interpretable images, five demonstrated total lesion glycolysis that decreased by ≥50% (three with complete resolution), and the other patients were able to discontinue their other anti-granulomatous therapies without experiencing a clinically significant worsening of disease. In general, cutaneous manifestations improved to a greater extent than the other organ disease manifestations; however, as the authors note, tofacitinib led to overall disease control that had not been previously achieved via alternative regimens. Their work demonstrated a significant decrease in Th1-mediated markers of inflammation. 

It is important to note that there is both specificity and redundancy in JAK-STAT inhibition with varying activity against individual cytokines based on the inhibition of individual or combination JAKs. Particularly relevant to sarcoidosis, IFN-γ signals via JAK1 and JAK2, which, in turn, activate STAT1, while IL-6 signals via JAK1, JAK2, and/or TYK2 and activates STAT3 [82]. Ultimately, all of the current FDA-approved JAK inhibitors—tofacitinib (JAK 1/3), ruxolitinib (JAK1/2), barcitinib (JAK 1,2), and upadacitinib (JAK 1)—have variable but clinically relevant targets [83]. 

#### 2.3.3. Adverse Effects and Clinical Monitoring 

In the preliminary report of the ongoing open-label clinical trial evaluating tofacitinib efficacy in refractory cutaneous sarcoidosis, the therapy was well tolerated, and there were no significant or dose-limiting adverse events [81]. Nevertheless, all JAK inhibitors carry risks of infection, cytopenia, and hyperlipidemia [73]. Opportunistic infections are likely the most common adverse effect associated with tofacitinib. The reactivation of tuberculosis was the most common infection observed in one review [84]. Increased rates of herpes zoster have also been observed [85]. The use of tofacitinib has been associated with lower gastrointestinal tract perforation [86]. There is also evidence of an increased risk of venous thromboembolism (VTE) in patients with cardiovascular risk factors or elevated VTE risk at baseline [87]. Additionally, treatment with tofacitinib conveyed an increased risk of major adverse cardiovascular events (MACE), including MI, cardiovascular death and stroke, in a cardiovascular risk-enriched population with rheumatoid arthritis [88].

Clinical monitoring generally involves routine evaluation for cytopenia, hyperlipidemia, hypertension, transaminitis, and infection. 

### 2.4. Anti-IL6 

#### 2.4.1. Rationale 

Elevations in the pleiotropic pro-inflammatory cytokine IL-6 have been observed in the serum and BAL fluid of patients with active pulmonary sarcoidosis since the 1990s [89,90,91]. Produced primarily by innate immune cells (including macrophages and dendritic cells) in response to local infection or tissue injury and signaling primarily via JAK/STAT and MAPK pathways, IL-6 functions as a systemic alert signal and helps to orchestrate the ensuing adaptive immune response [92,93]. Among other functions, it is known to promote (in conjunction with tissue growth factor-beta TGF-β) the differentiation of Th17 and Th17.1 effector T cells, which produce IL-17 and IFN-γ, respectively, which are both recognized as key cytokines involved in granuloma formation [94]. Additionally, it exerts an inhibitory effect on regulatory T cells (Tregs), which are responsible for reigning in the inflammation promulgated by effector T lymphocytes [95]. This exaggerated Th17 immune response (and the imbalanced Th17/Treg cell ratio) has been well described in the sera and BAL fluids of patients with sarcoidosis [96,97]. 

Additionally, early studies of IL-6 demonstrated its key role in inducing the hepatic production of acute phase reactants, including C-reactive protein (CRP), fibrinogen, haptoglobin, and serum amyloid A (SAA) [98]. Work by Chen, Moller, and colleagues has identified SAA, which is notably abundant in the granulomas of sarcoidosis, as a potential key mediator that perpetuates the production of cytokines promoting chronic granulomatous inflammation through stimulation of Toll-Like Receptor-2 (TLR2) in the absence of acute replicating infection [99,100]. In addition to its role in upregulating Th17 pathways of inflammation, IL-6 blockade is proposed to mitigate granulomatous inflammation via the downregulation of this insoluble acute-phase protein [101]. 

#### 2.4.2. Clinical Evidence 

In 2019, Sharp and colleagues reported their experience in treating four patients with refractory, steroid-dependent pulmonary sarcoidosis with the anti-IL-6 receptor monoclonal antibody tocilizumab [101]. All four patients were noted to have had multiple years (decades in three cases) of persistent respiratory symptoms despite chronic prednisone use and various steroid-sparing therapies, including TNF-inhibitors. Within two months of tocilizumab initiation, all four patients reported significant symptomatic improvement and were able to decrease their daily prednisone dose: three patients achieved steroid reduction of 50% or more, but all four patients remained on steroids at daily doses of 5–10 mg of prednisone. Three of the four patients experienced improvement in measures of lung function. At the time of their report, two of the four patients remained on anti-IL6 therapy. Tocilizumab had been discontinued briefly in one patient due to an episode of bronchitis. It had been held indefinitely in two other patients (one of whom developed breast cancer, while the other developed peripheral neuropathy). 

Sarilumab, an IL-6 receptor monoclonal antibody, was recently evaluated in a phase II clinical trial for patients with refractory pulmonary sarcoidosis (NCT04008069). Of the fifteen patients enrolled, four discontinued due to the worsening of their sarcoidosis, and five experienced the worsening of CT chest imaging. Compared to placebo, patients treated with sarilumab had no meaningful improvement in endpoints, including flare-free survival, changes in pulmonary function tests, chest imaging, patient-reported outcomes, and laboratory values [102].

#### 2.4.3. Adverse Effects and Clinical Monitoring 

The IL-6 receptor antibodies tocilizumab and sarilumab have gained FDA approval for use in the treatment of refractory rheumatoid arthritis [103]. They are generally well tolerated and have a safety profile that is comparable to those of other immunosuppressive therapies. They carry a risk of infection, including the reactivation of latent infection. The most common adverse reactions observed with intravenous monotherapy include upper respiratory tract infection, nasopharyngitis, headache, hypertension, and an increase in serum alanine aminotransferase. Additionally, studies have shown an elevated risk of lower intestinal perforation in patients with rheumatoid arthritis treated with tocilizumab, particularly those with a history of prior diverticulitis [104]. 

### 2.5. Anti-IL-1 

#### 2.5.1. Rationale 

IL-1 is often considered the prototypical pro-inflammatory cytokine, and efforts to curtail its activity in acute and chronic inflammatory conditions have been undertaken [105]. Along with TNF-α, IL-1 is released by activated macrophages in response to pathogen and damage-associated molecular patterns, and it is known to signal via the nuclear transcription factor NF-*k*B, a target of glucocorticoid therapy, thus drawing attention as a possible integral component in the inflammatory cascade that culminates in granuloma formation [4]. Early in vitro studies demonstrated that this Th1-cytokine (previously known as lymphocyte-activating factor) was produced in high quantities by the activated AM of patients with pulmonary sarcoidosis, and evidence of high-intensity alveolitis is shown in [106]. As previously described, an experiment by Müller-Quernheim and colleagues demonstrated higher spontaneous release of IL-1 and TNF-α by AM from patients with active sarcoidosis compared to inactive disease [23]. In 2000, Mikuniya and colleagues showed that the ratio of IL-1 receptor antagonist and IL-1β in the BAL fluids of patients with pulmonary sarcoidosis was positively correlated with improvements in chest radiograph and vital capacity, as well as negatively correlated with markers of disease activity, such as serum ACE levels [107]. In 2009, Wiken and colleagues demonstrated increased expression of pattern recognition receptors (specifically toll-like receptors 2 and 4) on the PBMCs of patients with sarcoidosis compared to healthy controls and a corresponding higher secretion of TNF-α and IL-1β when these TLRs were stimulated [108]. TNF-α and IL-1β have also been shown to increase the alveolar macrophage production of the chemokine ligand-20 (CCL20), which recruits dendritic cells, B cells, and T cells to the lungs [109,110]. 

#### 2.5.2. Clinical Evidence 

Canakinumab, a monoclonal antibody directed against IL-1β, was recently studied in a phase II placebo-controlled trial to evaluate its safety and efficacy for treating patients with pulmonary sarcoidosis (NCT02888080). Unfortunately, this trial explicitly excluded patients identified as having refractory disease. The posted results show no statistically significant difference in the primary outcome, which was FVC at 24 weeks. Anakinra, a recombinant human IL-1 receptor antagonist that is approved for use in rheumatoid arthritis, is currently being evaluated for use in cardiac sarcoidosis (NCT04017936) [111]. 

#### 2.5.3. Adverse Effects and Clinical Monitoring 

In the aforementioned phase II placebo-controlled trial of canakinumab in patients with pulmonary sarcoidosis (NCT02888080), three serious adverse events were reported among 20 patients receiving treatment (fewer than were observed in the corresponding placebo group), and 15 non-serious events were reported (14 in the placebo group). When used to treat non-sarcoidosis illnesses, the most commonly described adverse events associated with canakinumab include injection site reaction, gastrointestinal symptoms, rash, headache, and infection [112,113]. Patients should be evaluated for latent tuberculosis prior to the initiation of therapy. 

A large clinical trial of anakinra in a rheumatoid arthritis population revealed the most frequent adverse events to be injection site reactions and URIs. Serious infections were higher in the treatment group than the placebo group but were relatively low (5.37 events/100 patient years) [114]. Monitoring usually includes routine testing for neutropenia and changes in kidney function. 

### 2.6. Neuropilin-2 Immunomodulator 

#### 2.6.1. Rationale 

An emerging novel therapy for sarcoidosis sits at the intersection of two independent avenues of therapeutic investigation for immune-mediated disease: the proposed immune-mitigating effects of extracellular aminoacyl-transfer RNA (tRNA) synthetases and a class of receptors known as neuropilins. 

Aminoacyl-tRNA (tRNA) synthetases are enzymes essential for intracellular protein synthesis [115]. Autoantibodies to these enzymes, including anti-histidyl-tRNA synthetase (commonly known as anti-Jo-1), represent a key feature of anti-synthetase syndrome, characterized by the co-occurrence of immune cell-mediated interstitial lung disease and myositis [116,117]. There is evidence that extracellular fragments of these enzymes play a role in regulating innate and adaptive immunity [118,119,120]. Adams and colleagues recently confirmed that extracellular histidyl-tRNA synthetase is present in the sera of healthy humans but absent in patients with anti-Jo-1 positive anti-synthetase syndrome [121]. Their murine models suggest that the enzyme exerts an inhibitory effect on effector T cell activation, while the depletion of the enzyme through antibody neutralization augments immune-mediated inflammation [121]. 

Neuropilins (NRPs) are non-tyrosine kinase transmembrane glycoproteins expressed on the surface of many cells, including macrophages, dendritic cells, and T lymphocytes [122]. Originally identified as a coreceptor for vascular endothelial growth factor (VEGF) and class III semaphorins, early studies of NRPs elaborated their role in neural development and angiogenesis. Additionally, an important function in innate and cellular immunity has emerged. NRP1 expressed on the surface of myeloid dendritic cells (DCs) have been shown to facilitate their migration to lymphatics following antigen exposure and appear to be integral to the primary immune synapse of DCs with T-cells, thus initiating the process of antigen presentation [123,124]. Certain tumor-associated macrophages, which have been found to enable tumor progression by promoting angiogenesis and immune tolerance, are recruited to the hypoxic core of the tumor via the expression of NRP1 [125]. Both NRP1 and NRP2 are expressed on alveolar and bronchial macrophages, and there are some data to suggest that there is increased NRP2 expression on macrophages found within granulomas [126,127]. Efzofitimod (ATYR1923) is a first-in-class immunomodulator composed of a splice-variant of histidyl-tRNA synthetase, the sole binding partner of which is NRP-2 [127].

#### 2.6.2. Clinical Evidence 

A phase I/II randomized, double-blinded, and placebo-controlled trial evaluating the safety and efficacy of efzofitimod was recently published [128]. In this trial, 37 patients with pulmonary sarcoidosis (median disease duration, 4.2 years; range, 0.5–28 years) were randomized in a 2:1 fashion such that 25 patients received the study drug at various doses (1, 3, and 5 mg/kg per day). All patients were receiving corticosteroids at baseline (mean prednisone equivalent dose 13.2 ± 4.4 mg/day). The study population did not necessarily represent a refractory patient cohort, as more than 60% had received no additional therapies. While the primary outcome of the study was concerned with safety and tolerability, the authors reported a dose-dependent trend towards decreased steroid dependence over the 24-week trial period, specifically observing a 58% reduction in corticosteroid dose from baseline in the 5 mg/kg/day arm compared to a 48% reduction in the placebo group. Notably, three patients in the 5 mg/kg/day treatment group were able to be completely weaned from steroids with sustained remission, whereas no patients in any other treatment group exhibited sustained steroid-free remission. The two highest dose groups also exhibited improvement in lung function (percentage of predicted FVC and diffusing capacity of the lungs for carbon monoxide, DLCO) that did not reach statistical significance but was maintained throughout all time intervals of the study [128]. These findings support further investigation of this therapy with a larger study population and perhaps in a treatment refractory cohort. 

#### 2.6.3. Adverse Effects and Clinical Monitoring 

In this phase I/II clinical trial, efzofitimod was tolerated at all tested doses and deemed safe. Among the patients treated with the study drug, three Grade 3 (nonserious) adverse events were observed (depression, toothache, and myalgias), none of which were deemed to be likely related to the study drug. The only serious treatment-emergent adverse event (acute cholecystitis) observed among patients in the treatment arm was similarly deemed to be unlikely related to the study drug. One patient discontinued efzofitimod (at 1 mg/kg) due to alopecia, which was deemed to be likely related to the study drug [128].

### 2.7. mTOR Inhibitor

#### 2.7.1. Rationale 

Abnormal macrophage aggregation represents a key step in all granulomatous disease, including sarcoidosis. The mammalian target of the rapamycin (mTOR)-signaling pathway regulates macrophages, as well as monocytes and dendritic cells, via a metabolic checkpoint kinase, i.e., the mTOR complex 1 (mTORC1) [129]. A study by Linke and colleagues demonstrated that the activation of mTORC1 in macrophages induced the hypertrophy and proliferation of macrophages in mice, leading to granuloma formation [130]. 

#### 2.7.2. Clinical Evidence 

In 2020, Gupta and colleagues reported their experience in treating a patient with pulmonary sarcoidosis who was unable to taper below 15 mg of prednisone per day [131]. Following 10 months of treatment with the mTOR inhibitor sirolimus at 2 mg per day, the patient experienced symptomatic and radiographic improvement. A study of a large solid organ transplant population evaluated incident sarcoidosis among patients receiving mTOR inhibitors compared to calcineurin inhibitors [132]. There was no incident sarcoidosis among patients treated with mTOR inhibitors (compared to 0.2% incidence in the calcineurin-treated patients). 

#### 2.7.3. Adverse Effects and Clinical Monitoring 

Experience with sirolimus for the treatment of other indications (solid organ transplant, lymphangioleiomyomatosis, vascular anomalies) suggests that the most adverse effects may vary based on indication, but they commonly include peripheral edema, diarrhea, nausea, hypercholesterolemia, and bone marrow toxicity, as shown in [133,134]. Routine monitoring usually uses metabolic panels to assess for renal dysfunction and dyslipidemia and complete blood counts for cytopenias. 

### 2.8. GM-CSF Inhibitor

#### 2.8.1. Rationale 

Granulocyte-macrophage colony-stimulating factor (GM-CSF) is a cytokine involved in the recruitment of innate immune cells with an implicated role in a variety of autoimmune and inflammatory diseases [135]. It has been observed in the BAL fluid and serum of patients with pulmonary sarcoidosis and is generally associated with a chronic or progressive phenotype [136,137,138]. In vitro studies have demonstrated an exaggerated amount of TNF-α and IL-1β secreted by AMs and peripheral monocytes following GM-CSF stimulation in peripheral blood obtained from patients with sarcoidosis compared to healthy controls [139]. 

#### 2.8.2. Clinical Evidence 

A phase II randomized placebo-controlled study is currently evaluating the efficacy and safety of namilumab, a monoclonal antibody directed against GM-CSF, in patients with chronic pulmonary sarcoidosis (NCT05314517). Preliminary results from this trial have not yet been published.

#### 2.8.3. Adverse Effects and Clinical Monitoring 

Namilumab has been studied in refractory rheumatoid arthritis [140]. The most commonly listed adverse events were nasopharyngitis, dyspnea, bronchitis, and headache.

### 2.9. Anti-Fibrotic Therapy

#### 2.9.1. Rationale 

Advanced and end-stage pulmonary sarcoidosis is characterized by progressive pulmonary fibrosis and loss of lung function. Importantly, fibrotic changes occur at the sites of long-standing active granulomatous inflammation and typically exhibit histopathological features that are distinct from those of usual interstitial pneumonia (UIP) [141,142,143]. 

#### 2.9.2. Clinical Evidence 

The results of the Inbuild Trial support the use of antifibrotics to preserve lung function among patients with a progressive fibrosing phenotype of interstitial lung disease other than idiopathic pulmonary fibrosis [144]. Fewer than 10% of patients included in this trial carried a diagnosis of sarcoidosis, but there are no rigorous trials evaluating the use of anti-fibrotics to prevent progressive fibrosis in this population. A cogent case can be made that many patients with fibrosing pulmonary sarcoidosis can be controlled via anti-granulomatous therapy [145]. There is an ongoing placebo-controlled trial assessing the effectiveness of pirfenidone use in progressive fibrotic sarcoidosis (NCT03260556). 

#### 2.9.3. Adverse Events and Clinical Monitoring 

Safety data from clinical trials of pirferidone in patients with idiopathic pulmonary fibrosis (IPF) reveal that the drug is generally well tolerated, with the most commonly encountered adverse events being gastrointestinal symptoms and photosensitivity [146]. The drug can also be associated with transaminitis, and the routine monitoring of liver function tests is recommended. 

## 3. Conclusions

To date, clinical data remain limited for guiding therapeutic decisions for the management of refractory pulmonary sarcoidosis. This is further confounded by the lack of FDA approval of therapies beyond steroids for a sarcoidosis indication. Nevertheless, there are multiple promising classes of medications currently being used or under investigation for patients with chronic, progressive disease. The use of these therapies is informed by our expanding understanding of the pathobiology of the granuloma and phenotypes of disease, and emerging data from small trials and case series are promising.

**Table 1:** Current and Emerging Third Line Therapies for Sarcoidosis.

Therapy	Clinical Evidence	Route	Side Effects	Clinical Monitoring
TNF Inhibitors				
Infliximab	RTCs [34,35]	IV	Hypersensitivity reaction, infection, paradoxical adverse events, hepatotoxicity	Initial: hepatitis serologies, TB screen
				Ongoing LFT, CBC
Adalimumab	Case series [39]	SQ	Hypersensitivity reaction, infection, paradoxical adverse events, hepatotoxicity	Initial: hepatitis serologies, TB screen
	Clinical trial [40]			Ongoing: LFT, CBC
Anti-CD20				
Rituximab	Case series [59]	IV	Infusion reaction, infection, severe COVID-19 infections, PML	Initial: hepatitis serologies, TB screen
	Clinical trial [60]			Ongoing: CBC, IgG levels
JAK Inhibitor				
Tofacitinib	Case report [77]	PO	Infection, Cytopenia, Hyperlipidemia^,^ GI perforation, VTE, Diarrhea, hypertension, major adverse cardiovascular events, infection	Initial: TB screen, hepatitis serologies
	Ongoing clinical trial (NCT03910543)			Ongoing: CBC, BMP, LFT, lipid panel
Baricitinib	Case report [79]	PO	Infection, Cytopenia, Hyperlipidemia, GI perforation, VTE, Infection	Initial: TB screen, hepatitis serologies
				Ongoing: CBC, BMP, LFT, lipid panel
Ruxolitinib	Case report [78]	PO	Hypertension, hyperlipidemia, cytopenias, GI distress, dizziness, elevated aminotransferases, cough, dyspnea, muscle pain, fever	Initial: TB screen,
				Ongoing: CBC, LFT, lipid panel, BMP, blood pressure
Anti-IL6				
Tocilizumab	Case series [101]	IV/SQ	Hypersensitivity reaction, infection, headache, hypertension, constipation, hyperlipidemia, GI tract perforation	Initial: TB screen, lipid panel at baseline and 4–8 weeks after initiation
				Ongoing LFT, CBC
Anti-IL6 Receptor				
Sarilumab	Ongoing clinical trial (NCT04008069)	SQ	Hypersensitivity reaction, infection, headache, hypertension, constipation, hyperlipidemia, GI perforation	Initial: TB screen, lipid panel at baseline and 4-8 weeks after initiation
				Ongoing: LFT, CBC
Neuropilin 2 Immunomodulator				
Efzofitimod	Ongoing clinical trial (NCT05415137)	IV	Under investigation	Under investigation
Anti-IL1β				
Canakinumab	Ongoing clinical trial (NCT02888080)	SQ	Gout flares, diarrhea, nausea, abdominal pain, cytopenias, injection site reaction, headache, muscle cramps	Initial: TB screen
				Ongoing: CBC
Anti-IL1				
Anakinra	Ongoing clinical trial (NCT04017936)	SQ	Infection, injection site reaction, headache, arthralgias	Initial: TB screen
				Ongoing: CBC
mTOR inhibitor				
Sirolimus	Case report [131]	PO	Edema, hyperlipidemia, diarrhea, cytopenias, arthralgias, increased serum creatinine	Lipid panel, urine protein creatinine ratio, BMP, CBC, serum drug level, blood pressure
Anti-GM-CSF				
Namilumab	Ongoing clinical trial (NCT05314517)	SQ	Under investigation	Under investigation
Anti-fibrotic				
Pirfenidone	Ongoing clinical trial (NCT03260556)	PO	Rash, abdominal pain, diarrhea, anorexia, nausea, vomiting, fatigue, dizziness, URI, increased aminotransferases	LFT

Abbreviations: BMP basic metabolic panel; CBC complete blood count; CD cluster of differentiation; GI gastrointestinal; GM-CSF granulocyte-macrophage colony-stimulating factor; JAK janus kinase; IL interleukin; IV intravenous; LFT liver function test; mTOR mammalian target of rapamycin; PML progressive multifocal leukoenceophalopathy; PO per os (by mouth); Ref reference cited in the text; RTC randomized control trial; SQ subcutaneous; TB tuberculosis; TNF tumor necrosis factor; URI upper respiratory tract infection; VTE venous thromboembolism.

## Data Availability

No new data were created or analyzed in this study. Data sharing is not applicable to this article.
